# Heavy Metal contamination of Dietary Supplements products available in the UAE markets and the associated risk

**DOI:** 10.1038/s41598-020-76000-w

**Published:** 2020-11-02

**Authors:** Ammar Abdulrahman Jairoun, Moyad Shahwan, Sa’ed H. Zyoud

**Affiliations:** 1 Discipline of Social and Administrative Pharmacy, School of Pharmaceutical Sciences, Universiti Sains Malaysia, George Town, Pulau Pinang Malaysia; 2 College of Pharmacy and Health Sciences, Ajman University, Ajman, UAE; 3 Poison Control and Drug Information Center (PCDIC), College of Medicine and Health Sciences, An-Najah National University, Nablus, 44839 Palestine; 4 Clinical Research Centre, An-Najah National University Hospital, Nablus, 44839 Palestine

**Keywords:** Environmental sciences, Environmental social sciences

## Abstract

A specific safety concern is the possibility that a dietary supplement could be contaminated with heavy metals. This research was undertaken to investigate the daily exposure levels of heavy metals in dietary supplements available in the UAE and to explore the factors associated with the contamination of dietary supplements with heavy metals. A total of 277 dietary supplement samples were collected from the UAE market and prepared for the analysis of selected heavy metal contamination. Inductively coupled plasma mass spectrometry (ICP-MS) was used to determine the presence of heavy metals. The average daily intake of cadmium was 0.73 μg [95% CI 0.61–0.85], compared to the acceptable daily intake (ADI) of 6 μg; the daily intake of lead was 0.85 μg [95% CI 0.62–1.07], compared to the acceptable daily intake (ADI) of 20 μg; and the daily intake of arsenic was 0.67 μg [95% CI 0.57–0.78], compared to the acceptable daily intake of 10 μg. Although the dietary supplements available in the UAE have low levels of heavy metal contamination, numerous individuals are consuming a number of different dietary supplements every day and thereby may experience a cumulative level of toxic exposure. Dietary supplements formulations (Categories), dosage forms and country of origin are strong determents of heavy metal contamination in dietary supplements products.

## Introduction

Across the world, supplement products are designated as dietary, food, or health supplements, and various national authorities and regulatory regimes are responsible for controlling and monitoring them. They are placed in the category of dietary supplements in the USA and come under the purview of the Food and Drug Administration (FDA); they come under the jurisdiction of the European Food Safety Authority (EFSA) in Europe, where they are classified as food supplements; in Canada, Health Canada is responsible for monitoring, and they are classified as natural health products. In the UAE, the Dubai Municipality Health and Safety Department places these products in the category of health supplements (HS) and offers a definition of them as products (except for tobacco) intended to complement the diet and that include one or a combination of dietary ingredients, e.g., amino acids, herbs/botanicals, minerals, or vitamins^1^. In the USA, there are approximately 55,000 different dietary supplements available, with approximately 60% of Americans using them^2^. In Europe, approximately 50% of people use natural herbal products (NHPs), as do approximately 71% of Canadians (with 38% using them daily)^3,4^. There are numerous claims and beliefs around the efficacy of herbal medication, but reports have arisen of acute/chronic toxicity caused by their usage^5^. As these traditional remedies have become more popular and more readily available, concern has arisen with respect to their safety and efficacy and to the level of responsibility of the practitioners in this field. It has been shown that traditional Asian and Indian remedies can be high in mercury, lead, and arsenic (Asian remedies)^5^ or high levels of lead only (Indian remedies)^6^. Cases of poisoning attributable to toxic metals being present in medicinal plants have been reported in the USA, Europe, and Asia^7–10^. A toxicology unit based in London released a case series from 1991 to 1995 of adverse occurrences linked to traditional medicines^11^. Out of a dozen cases of mercury, arsenic, or lead poisoning, 75% had associations with Indian herbal remedies. There have been many case reports/series related to heavy metal poisoning attributable to traditional Chinese medicine (TCM)^12^; lead is the most frequently cited culprit, but there have also been cases of thallium, copper, arsenic, cadmium, and mercury found in TCM^12^. Officials in California have undertaken the screening of imported Chinese remedies being offered for sale in Californian herbal outlets, looking for heavy metals and undeclared pharmaceuticals. Out of 251 products tested, 23 had multiple contaminants/adulterants, 36 held an average of 14.6 ppm arsenic, 24 had a minimum of 10 ppm lead, and 35 held an average of 1046 ppm mercury^13^. Additional research by Koh and Woo showed that 42 proprietary Chinese medicines contained toxic heavy metals at levels in excess of the legal limits for Singapore^14^. In adults, lead poisoning can increase blood pressure, cause cardiovascular disease, delay cognitive development, and cause renal tumours. Lead has a severe impact on the human brain; this seems to be the case in children in particular, with lead exposure being associated with poor learning capability and lower IQs^15–18^. Organic mercury has a higher toxicity than does inorganic mercury, as it is easier to ingest; it is highly damaging to the development of both foetuses and children^19^. High levels of exposure to either inorganic or organic mercury can provoke neurological problems, which include seizures and even mortality^15^. Excessive ingestion of cadmium chiefly attacks the kidneys and, to a lesser degree, the reproductive system^15^, with arsenic having been shown to cause cancers^20^, impaired reproductive systems^21^, and atherosclerosis^22^. Furthermore, chronic cadmium exposure can lead to nephrotoxicity in humans, chiefly as a result of tubular reabsorption abnormalities^23^. Mercury and lead can be damaging to the nervous and renal systems and are capable of crossing the placental barrier and poisoning the foetus^24,25^.

In accordance with the legislation in the United Arab Emirates, manufacturers of dietary supplements are legally required to provide documented proof that the products they produce are manufactured in a safe way and are tested to ensure they are pure and of a satisfactory quality. However, despite such rules, a myriad of cases have arisen in which health supplements that have later been found to contain hazardous heavy metals have been sold on the open market^23^. In addition, the products are not supported by solid clinical trials or suitable documentation^26,27^. In many cases, dietary supplements have been removed from sale and recalled after tests have found they were contaminated with microorganisms or prescription drugs^28–30^. Another significant challenge that impacts the safety of DS concerns the fact that many of them are manufactured in different countries. For example, they may be manufactured in China for sale in the United States. This can result in variations in the purity and quality of the products and the regulations by which they are produced and monitored. Furthermore, a lack of standardization between origin and production can entail that there are variations between batches. To our knowledge, limited research has been conducted in the UAE concerning the heavy metal contamination in dietary supplements.

Thus, this research aims to carry out an evaluation of the heavy metal content/contamination in dietary supplements offered in the UAE market and to determine the significant factors associated with such content which could be help the regulatory bodies in structuring a risk assessment module for the safety of dietary supplements.

## Results

### Sample baseline characteristics

Table 1 presents the sample baseline characteristics of the dietary supplements (DSs). A total of 277 DS samples were collected from the UAE market and prepared for analysis of selected heavy metal contamination. The categories and ingredients of the studied DSs were as follows: 121 (43.7%) were herbal/botanical supplements; 19 (6.9%) vitamins; 5 (1.8%) minerals; 23 (8.3%) vitamins and minerals; 9 (3.2%) vitamins and herbs; 26 (9.4%) minerals and herbs; 30 (10.8%) vitamins, minerals and herbs; 13 (4.7%) herbs, vitamins, minerals, fatty acids and enzymes; and 20 (7.2%) herbs, probiotics, enzymes and fatty acids. The dosage forms of the DS sample also varied. Approximately 13 (4.7%) were powders/liquids, 71 (25.6%) tablets, 23 (8.3%) soft gels, 157 (56.7%) capsules and 13 (4.7%) caplets. Of the total, 168 (60.6%) samples were made in the USA, 25 (9%) were made in India, 28 (10.1%) were made in Canada, and 8 (2.9%) were made in Australia.

**Table 1:** Number and percentages of sample baseline characteristics.

Characteristics	Groups	Frequency	Percentage
DS combination of ingredients	Herbal/botanical supplements	121	43.7
	Vitamins	19	6.9
	Minerals	5	1.8
	Vitamins + minerals	23	8.3
	Vitamins + herbs	9	3.2
	Minerals + herbs	26	9.4
	Vitamins + minerals + herbs	30	10.8
	Herbs + vitamins + minerals + fatty acids + enzymes	13	4.7
	Herbs + probiotics + enzymes + fatty acids	20	7.2
	Amino acids + collagen	11	4
Dosage forms	Powder/liquid	13	4.7
	Tablet	71	25.6
	Soft gel	23	8.3
	Capsule	157	56.7
	Caplet	13	4.7
Country of origin	USA	168	60.6
	India	25	9
	EU	48	17.3
	Canada	28	10.1
	Australia	8	2.9

### Assessment of daily intake (μg) of heavy metals from dietary supplement products and acceptable daily intake (ADI)

Estimates of the daily intake of the three heavy metals are summarized in Table 2. The average daily intake of cadmium was 0.73 μg [95% CI 0.61–0.85], compared to the acceptable daily intake (ADI) of 6 μg; the daily intake of lead was 0.85 μg [95% CI 0.62–1.07], compared to the acceptable daily intake (ADI) of 20 μg; and the daily intake of arsenic was 0.67 μg [95% CI 0.57–0.78], compared to the acceptable daily intake of 10 μg. Assuming that the consumers of the dietary supplements adhere to the manufacturer’s instructions for use, the daily intake of arsenic would not exceed the ADI of any of the 277 DS products. However, the daily intake of cadmium exceeded the ADI for two products, and the daily intake of lead exceeded the ADI for one product. Overall, three dietary supplement samples (1.1%) exceeded the ADI of cadmium, lead and arsenic.

**Table 2:** Results of daily intake (μg/g) of heavy metals from dietary supplement products compared to acceptable daily intake (ADI).

Heavy metal	ADI (μg)	Daily intake					
		Mean	± SD	95% CI		Median	Q3–Q1
Cadmium (Cd)	6.00	0.73	± 1.03	0.61	0.85	0.47	0.84–0.18
Lead (Pb)	20.00	0.85	± 1.94	0.62	1.07	0.52	0.88–0.25
Arsenic (As)	10.00	0.67	± 0.89	0.57	0.78	0.49	0.81–0.21

### Daily intake (μg) of heavy metals from dietary supplement products according to sample baseline characteristics

The distribution of the daily intake of heavy metals from the dietary supplement products according to baseline characteristics is shown in Table 3.

**Table 3:** Daily intake (μg) of heavy metals from dietary supplement products according to sample baseline characteristics.

DS categories	Groups	Cd daily intake			Pb daily intake			As Daily intake		
		Mean	± SD	*P* value	Mean	± SD	*P* value	Mean	± SD	*P* value
	Herbal supplements	0.65	0.81	< 0.001	0.88	2.72	0.666	0.69	1.02	< 0.001
	Vitamins + minerals	0.41	1.26		0.53	1.20		0.19	0.28	
	Vitamins + minerals + herbs	0.83	1.04		0.81	0.66		0.74	0.60	
	Amino acids + collagen	0.19	0.33		0.76	1.21		0.65	1.04	
	Herbs + vitamins + minerals + fatty Acids + enzymes	1.36	0.97		1.13	0.61		0.98	0.55	
	Herbs + probiotics + enzymes + fatty acids	1.51	1.27		1.40	1.21		1.40	1.21	
Dosage forms	Powder/liquid	1.14	1.80	0.030	1.52	1.92	0.164	2.17	2.77	< 0.001
	Tablet	0.81	1.16		1.12	3.55		0.57	0.52	
	Soft gel	1.22	1.33		1.29	1.32		1.24	1.31	
	Capsule	0.60	0.82		0.61	0.53		0.54	0.40	
	Caplet	0.50	0.34		0.77	0.68		0.48	0.35	
Country of origin	USA	0.76	1.02	0.41	0.74	0.83	0.007	0.76	1.04	0.082
	India	0.97	1.66		2.19	5.7		0.70	0.73	
	EU	0.54	0.88		0.58	1.1		0.40	0.41	
	Canada	0.78	0.56		0.86	0.66		0.77	0.55	
	Australia	0.39	0.19		0.56	0.62		0.24	0.20	

There was a significant association between the daily intake of cadmium and dietary supplement categories (*P* < 0.001). The daily intake of cadmium from dietary supplements increased with the following: DS products containing herbs, probiotics, enzymes and fatty acids; DS products containing herbs, vitamins, minerals, fatty acids and enzymes; and DS products containing vitamins, minerals and herbs. Similarly, we detected a statistically significant association between the daily intake of cadmium and dosage forms (*P* = 0.030). The daily intake of cadmium from dietary supplements increased with powder/liquid dietary supplements and soft-gel dietary supplements.

Moreover, we reported a statistically significant relation between the country of origin and the daily intake of lead (*P* = 0.007). The daily intake of lead from dietary supplements was higher in products made in India and the EU.

In addition, we observed a statistically significant association between the daily intake of arsenic and dietary supplement categories (*P* < 0.001). The daily intake of arsenic from dietary supplements increased with the following: DS products containing herbs, probiotics, enzymes and fatty acids; and DS products containing herbs, vitamins, minerals, fatty acids and enzymes. The same pattern of results was reported between the daily intake of arsenic and dosage forms (*P* < 0.001). The daily intake of arsenic from dietary supplements increased with the powder/liquid dietary supplements and soft-gel dietary supplements. For more details, see Table 3.

### Factors associated with dietary supplement contamination with (μg) heavy metals

Univariate and multivariate logistic regression analysis was used to examine the factors associated with dietary supplement contamination with heavy metals.

From the multivariate liner regression model, dietary supplements containing herbs, vitamins, minerals, fatty acids and enzymes (β = 0.725, CI 0.168–1.282, *P* value = 0.011) and supplements containing herbs, probiotics, enzymes and fatty acids (β = 0.876, CI 0.421–1.33, *P* value = 0.000) jointly increased the risk of dietary supplement contamination with cadmium (Table 4).

**Table 4:** Univariate and multivariate analysis of factors associated with Cd daily intake.

Factors	Cd daily intake (μg)							
	Univariate				Multivariate			
	β	95% CI		*P* value	β	95% CI		*P* value
Amino acids + collagen	Ref				–	–	–	–
Herbal supplements	0.46	− 0.15	1.073	0.14	–	–	–	–
Vitamins + minerals	0.22	− 0.43	0.88	0.49	–	–	–	–
Vitamins + minerals + herbs	0.64	0.01	1.27	0.048	–	–	–	–
Herbs + vitamins + minerals + fatty acids + enzymes	1.17	0.37	1.96	0.004	0.725	0.168	1.282	0.011
Herbs + probiotics + enzymes + fatty acids	1.32	− 0.39	0.78	< 0.001	0.876	0.421	1.331	< 0.001
Caplet	Ref				–	–	–	–
Powder/liquid	0.63	− 0.015	1.42	0.11	–	–	–	–
Tablet	0.31	− 0.29	0.91	0.31	–	–	–	–
Soft gel	0.71	0.02	1.40	0.04	–	–	–	–
Capsule	0.10	− 0.48	0.68	0.73	–	–	–	–
Australia	Ref				–	–	–	–
USA	0.36	− 0.37	1.09	0.34	–	–	–	–
India	0.57	− 0.25	1.39	0.18	–	–	–	–
EU	0.14	− 0.63	0.91	0.72	–	–	–	–
Canada	0.38	− 0.43	1.19	0.36	–	–	–	–

*P* values less than 0.05 were considered statistically significant, “–” not included in the multivariate linear regression model.

β, beta coefficient; CI, confidence interval.

Regarding the lead, multivariate liner regression analysis showed that dietary supplements in capsule form (β = −0.75, CI − 1.21 to − 0.29, *P* value = 0.002) decreased the risk of lead contamination in dietary supplements, but supplements produced in India (β = 1.59, CI 0.83–2.37, *P* value = 0.000) increased the risk of dietary supplement contamination with lead (Table 5).

**Table 5:** Univariate and multivariate analysis of factors associated with Pb daily intake.

Factors	Pb Daily intake (μg)							
	Univariate				Multivariate			
	β	95% CI		*P* value	β	95% CI		*P* value
Amino acids + collagen	Ref				–	–	–	–
Herbal supplements	0.12	− 1.09	1.33	0.85	–	–	–	–
Vitamins + minerals	− 0.23	− 1.51	1.06	0.73	–	–	–	–
Vitamins + minerals + herbs	0.048	− 1.20	1.29	0.94	–	–	–	–
Herbs + vitamins + minerals + fatty acids + enzymes	0.37	− 1.20	1.94	0.64	–	–	–	–
Herbs + probiotics + enzymes + fatty acids	0.64	− 0.79	2.08	0.38	–	–	–	–
Caplet	Ref				–	–	–	–
Powder/liquid	0.75	− 0.74	2.242	0.32	–	–	–	–
Tablet	0.35	− 0.79	1.49	0.55	–	–	–	–
Soft gel	0.53	− 0.79	1.85	0.43	–	–	–	–
Capsule	− 0.16	− 1.25	0.94	0.78	− 0.75	− 1.21	− 0.29	0.002
Australia	Ref				–	–	–	–
USA	0.18	− 1.18	1.54	0.79	–	–	–	–
India	1.64	0.12	3.17	0.035	1.599	0.83	2.37	< 0.001
EU	0.03	− 1.40	1.46	0.97	–	–	–	–
Canada	0.30	− 1.20	1.80	0.69	–	–	–	–

*P* values less than 0.05 were considered statistically significant, “–” not included in the multivariate linear regression model.

β, beta coefficient; CI, confidence interval.

For arsenic, multivariate liner regression analysis showed that supplements containing vitamins and minerals (β = −0.55, CI − 0.79 to − 0.31, *P* value = 0.000) decreased the risk of dietary supplement contamination with arsenic, while supplements containing herbs, probiotics, enzymes and fatty acids (β = 0.53, CI 0.16–0.89, *P* value = 0.005), supplements in powder/liquid form (β = 1.65, CI 1.22 to 2.07, *P* value = 0.000) and supplements in soft-gel form (β = 0.52, CI 0.18 to 0.86, *P* value = 0.003) had an increased risk of contamination with arsenic (Table 6).

**Table 6:** Univariate and multivariate analysis of factors associated with As daily intake.

Factors	As daily intake (μg)							
	Univariate				Multivariate			
	β	95% CI		*P* value	β	95% CI		*P* value
Amino acids + collagen	Ref				–	–	–	–
Herbal supplements	0.04	− 0.49	0.56	0.89				
Vitamins + minerals	− 0.46	− 1.02	0.10	0.11	− 0.55	− 0.79	− 0.31	0.000
Vitamins + minerals + herbs	0.09	− 0.45	0.63	0.75	–	–	–	–
Herbs + vitamins + minerals + fatty acids + enzymes	0.33	− 0.35	1.01	0.34	–	–	–	–
Herbs + probiotics + enzymes + fatty acids	0.75	0.13	1.38	0.018	0.53	0.16	0.89	0.005
Caplet	Ref				–	–	–	–
Powder/liquid	1.69	1.07	2.32	< 0.001	1.65	1.219	2.075	< 0.001
Tablet	0.09	− 0.38	0.57	0.69	–	–	–	–
Soft gel	0.76	0.21	1.31	0.007	0.52	0.18	0.86	0.003
Capsule	0.06	− 0.40	0.51	0.81	–	–	–	–
Australia	Ref				–	–	–	–
USA	0.51	− 0.12	1.14	0.11	–	–	–	–
India	0.46	− 0.25	1.16	0.20	–	–	–	–
EU	0.15	− 0.51	0.82	0.65	–	–	–	–
Canada	0.52	− 0.17	1.22	0.14	–	–	–	–

*P* values less than 0.05 were considered statistically significant, “–-” not included in the multivariate linear regression model.

β, beta coefficient; CI, confidence interval.

## Discussion

In the UAE, only a small number of dietary supplement products are produced by a small number of companies; most of these types of products are imported from other countries. The dietary supplement market in the UAE is growing each year. In 2014, Dubai had 690 outlets for such products, increasing to 714 in 2015 and 800 in 2016^1^. Additionally, there has been a very significant increase in the numbers and total weights of dietary supplement consignments that have passed through Dubai ports and that have been subject to inspection by the health and safety department between 2012 and 2015. It is vital that their safety be critically evaluated to allay concerns regarding ways in which ready-made dietary supplements could be of poor quality, adulterated, and/or toxic. A specific safety concern is the possibility that a dietary supplement could be contaminated with heavy metals. This research was undertaken to investigate the daily exposure levels of heavy metals in dietary supplements available in the UAE market and to investigate why certain supplements should be contaminated with such metals.

This research demonstrated that across all products, those that did not comply with the recommended daily limits for cadmium, lead, and/or arsenic comprised 1.1% of the total [95% CI − 0.14 to 2.3], which is, on the whole, a reassuring and satisfactory outcome. The same pattern of the results were reported in other previous studies in which few dietary supplements products exceeded established daily limits for heavy metal contamination when taken on their own^31,32^.

This research found that the concentration of the average daily intake of cadmium was 0.73 μg/day, which exceeded that found by Canadian researchers (0.199 μg/day)^31^ but was less than that found in another study in the Emirates (0.83 μg/day)^32^. This research found an estimated average daily arsenic intake of 0.67 μg/day, far below the results in the Canadian study (21.7 μg/day)^31^ and in another study in the Emirates (0.92 μg/day)^32^. For lead, this research found an average daily intake of 0.85 μg/day, comparable to that found in the Emirates research (0.88 μg/day)^32^ but lower than that found in the Canadian research (1.49 μg/day)^31^.

The low levels of contamination of heavy metals in dietary supplements available in the UAE are attributable to the country's regulatory regime, in which health regulators and municipalities demand that all dietary supplements for sale in the UAE should be registered to ensure that they are safe, effective, and of good quality. Additionally, environmental controls and industrial hygiene have been improved so that it is less likely that the signs/symptoms of heavy metal poisoning are missed.

In this research, an exploration was undertaken of those factors that have a significant association with contaminated dietary supplements in terms of heavy metals to discover whether some dietary supplements available in the UAE represent a hazard to public health which could help the regulatory bodies in development of risk assessment module for dietary supplements products. Accordingly, dietary supplement formulations (categories) and dosage forms were significantly associated factors for dietary supplement contamination with heavy metal (Table 3).

It’s worthy to note that multivariate linear regression analysis was used in this study to identify the significant risk factors for heavy metal contamination in dietary supplements.

This research demonstrated that dietary supplements containing fatty acids, enzymes, minerals, vitamins, and herbs and those containing fatty acids, enzymes, probiotics, and herbs had the greatest risk of being contaminated with cadmium (Table 4). This may be because these products have a higher number of ingredients than other products have, or it may be attributable to the herbs that these products contain.

The outcomes of this research demonstrated that dietary supplements available in capsule form are less likely to be contaminated with lead, while supplements made in India had a high risk of being contaminated with lead (Table 5).

There are a number of reasons that may influence the greater likelihood of heavy metals being found in Indian-made dietary supplements. First, heavy metals may have been deliberately added to the ingredients to promote supposed medicinal benefits; some Indian medical practitioners believe that the human body requires zinc, tin, silver, mercury, iron, gold, copper, and/or lead for proper functioning^33^. Furthermore, certain Ayurvedic preparations contain notable levels of arsenic, lead, and/or mercury^34,35^. In this medical tradition, some practitioners believe that metals/metalloids ought to be added to minerals for optimal health balance. Thus, when supplements from this tradition contain metals, these metals may have been added deliberately (once it has undergone traditional cleansing) and not because of contamination^36,37^.

The outcomes of this research have demonstrated that when a supplement contains minerals and vitamins, it is less likely to be contaminated with arsenic, but those made from powders, liquids, or soft gels, as well as those containing herbs, enzymes, probiotics and fatty acids, are more likely to be contaminated with arsenic (Table 6).

These findings may be attributable to the fact that it is difficult to assure the quality of herbal medicinal products, as the finished product contains a large number of unidentified chemical elements, and generally, the specific bioactive components are not known^38^. Additionally, there is often no standardized process for harvesting or processing raw materials; thus, different batches of dietary supplements can vary significantly, which makes it difficult to produce an overall assessment of safety and/or efficacy^31^.

## Conclusion

Although the dietary supplements available in the UAE have low levels of heavy metal contamination, numerous individuals are consuming a number of different dietary supplements every day and thereby may experience a cumulative level of toxic exposure. Dietary supplements formulations (Categories), dosage forms and country of origin are strong determents of heavy metal contamination in dietary supplements products. Therefore, it is imperative to include these factors to develop a risk assessment module for dietary supplements products on regulatory level to ensure the quality and safety of dietary supplements products.

## Methods

### Sample calculation (sampling method)

Local business directories were examined to identify the various outlets selling dietary supplements in the UAE, specifically pharmacies, para-pharmacies, and nutrition/healthcare shops. In total, 1500 were noted, and an Excel spreadsheet was used to create a sampling framework containing the names, locations, emails and telephone numbers of these businesses. We then created the study sample by using a simple random selection method, choosing businesses on the basis of their ID numbers, which were layered according to location and type of business. A random selection from each chosen premise was made for a package of each dietary supplement meant to be taken orally, regardless of the country in which it was manufactured. The selection criteria for dietary supplement products was based on UAE, Dubai municipality Health and Safety Department definition as: any product intended to complement the diet and that include one or a combination of dietary ingredients, e.g., amino acids, herbs/botanicals, minerals, or vitamins (except for tobacco).

To obviate any problems with sample duplication and to allow tracking, all items were given a code reference number. These details were recorded for each sample: name of product, name brand, category of item, subcategory of item, batch number, barcode, size/volume, dosage form, recommended dosages, country of origin/manufacturer, and from where in each outlet the product was selected. If we found that a product was duplicated between two or more premises (i.e., a product with the same name, manufacturer, size/volume, barcode, and formulation), whichever sample that was selected first was used for testing, and the other products were returned. Any products with the same name but with different manufacturers or formulations (e.g., tablets/capsules) were regarded as individual products, and both went into the testing. Each product was sent to the laboratory to be tested on the day they were selected.

### Instrumentation and acquisition conditions

The presence of heavy metals was detected through the use of inductively coupled plasma mass spectrometry (ICP-MS, AGILENT, MODEL-7700X, YEAR—2011). The samples were prepared using microwave acid digestion (CEM, US, MODEL-MARS 5, YEAR—2011). They subsequently underwent multi-element determination through the use of collision/reaction cell (ICP-MS) in which helium (He) collision gases were employed to eliminate spectral interferences. The same method was then employed to measure all samples and elements in standard mode. During the process of evaluation and washout, an integrated sample introduction system (ISIS) was employed at a 0.1 rps pump speed. This reduced the quantity of matrix at the interface and enhanced the throughput of the sample. A standard sample introduction system that consisted of a quartz spray chamber, MicroMist glass concentric nebulizer, nickel interface cones, and a quartz torch with a 2.5-mm i.d. injector was utilized to configure the 7700 × ICP-MS^39^.

### Preparation of test portions

Tablets were crushed with a mortar and pestle. The crushed tablets were mixed thoroughly, and the whole oil capsules were digested. Containers of liquids were shaken vigorously before opening the container.

### Sample preparations

Approximately 1.0 ± 0.1 g of a homogeneous sample was added to 6 + 2 + 2 mL + 0.25 mL of a Suprapur nitric acid + hydrogen peroxide + hydrochloric acid + ISTD solution using a glass volumetric pipette and a micropipette in sealed clean Teflon microwave digestion vessels, and the sample was digested at 180 °C with a 10-min holding time. The solutions were diluted to a final volume of 50 mL with deionized water. Each solution was filtered through a 0.45 µm nylon filter, as required. The resultant solutions were subjected to elemental analysis by ICP-MS, and blank, duplicate, and spike samples were run for each digestion cycle.

### Calibration standards and internal standard

Calibration standards (0.2–50 µg/L) for As, Cd and Pb were prepared from 1000 µg/L single element standards (source: Supelco). An ISTD solution (50 µg/L) containing 100 µg/L of Sc, Rh, In and Ge was also prepared from a mixed element standard (source: Agilent). Calibration standards and ISTDs were prepared to match the acid content of the sample solutions. The ISTD solution was added to the sample using a calibrated micropipette.

### Validation methodology for quantitative procedures

The detection and quantification limits obtained using this method were 2 µg/L and 10 µg/L, respectively. Accurate spike recovery (between 80 and 120% of the spike value) was demonstrated for the quantitative procedures for the mean of 6 samples spiked at concentrations ranging from LOQ to medium and high levels of the calibration concentrations for each target element. In this validation procedure, we used 2.5 ppb, 250 ppb and 4000 ppb spikes of dietary supplement (external) samples that were prepared and analysed every 3 spike levels. Repeatability was demonstrated by external precision, and no more than a 20% RSD was found for 6 separate samples spiked at the indicated levels.

### Quality control and Quality Assurance (QC/QA) procedures

QC/QA procedures were undertaken during the course of developing the method. As a reagent blank, a sample blank spike was created for every digestion level, with a matrix spike being created every 15 samples. For intermediate standard checks, a standard solution was run following the linearity to verify the calibration curve employing an identical standard solution. Internal quality control standards (IQCs) were prepared from a variety of lot numbers and concentrations that were not incorporated in the linearity curve. Across the run, following every 10 to 15 samples, analysis of a calibration verification solution was undertaken. This must be done to measure system suitability. The following acceptance criteria were set:

- The reagent blank should be below the method detection limit;
- There should be less than a 20% relative difference between standards and duplicates;
- The sample blank spike/matrix spike percentage recovery should be between 70 and 120%; and for the intermediate check standard solution, the recovery percentage should fall between 80 and 120%.

### Limit of quantification (LOQ) and results reporting

The limit of quantification (LOQ) for the instrument is 50 μg/g for lead and arsenic and 10 μg/g for cadmium. For the assessment of reproducibility, all specimens containing any relevant metals when first tested underwent repeat testing. Additionally, retesting was undertaken with 10 randomly selected samples that had shown no signs of heavy metals. The acceptable daily intake (ADI) value was copied from the guidance of the American Herbal Products Association, which in turn is derived from the protocols of a number of national/international bodies^40^.

### Ethical considerations

Approval for the study was obtained from the Institutional Review Board of An-Najah National University (ref Phd/2/20/14).

### Statistical analysis

Data analysis was undertaken employing SPSS version 24 Chicago, IL, US. The qualitative variables were summarized using frequencies and percentages. With every product, the test concentration of the metal (μg/g) and the manufacturer's advised daily adult dose (g) were used to estimate how much of each metal would be ingested daily. We calculated the mean, median, and standard deviation for the daily consumption for each metal. One-way ANOVA and their nonparametric versions were employed to test the average daily intake of heavy metals for various groups. To explore the factors linked to dietary supplements being contaminated with heavy metals, univariate and multivariate linear regression models were employed. The criteria for a decision having statistical significance were a *P* value < 0.05 and a 95% confidence interval.

## Data Availability

Data is available from the corresponding author upon reasonable request.
